# The effects of a saffron extract (Affron®) on mood, sleep, self-esteem, and exploratory measures of physical appearance in women aged 50 to 70 years experiencing low mood and poor sleep: a randomised, double-blind, placebo-controlled trial

**DOI:** 10.3389/fnut.2026.1838513

**Published:** 2026-06-17

**Authors:** Adrian L. Lopresti, Stephen J. Smith

**Affiliations:** 1 Clinical Research Australia, Perth, WA, Australia; 2 College of Science, Health, Engineering and Education, Murdoch University, Perth, WA, Australia

**Keywords:** clinical trial, *Crocus sativus*, depression, physical attractiveness, saffron, self-esteem, sleep

## Abstract

**Background:**

Depression is commonly accompanied by sleep disturbances and low self-esteem. Saffron (*Crocus sativus* L.) has demonstrated antidepressant and sleep-enhancing effects in previous clinical trials; however, its efficacy in women experiencing both low mood and poor sleep has not been specifically examined. Furthermore, the effects of saffron on self-esteem and skin health remain largely unexplored.

**Objective:**

To examine the effects of supplementation with a saffron extract (Affron®) on mood, sleep, self-esteem, and skin health in women aged 50–70 years experiencing low mood and self-reported poor sleep.

**Methods:**

In this randomised, double-blind, placebo-controlled trial, 86 women were allocated in a 1:1 ratio to receive either 28 mg/day of a saffron extract or placebo for 12 weeks. The primary outcome was the Depression, Anxiety and Stress Scale–21 (DASS-21) depression subscale. Secondary outcomes included the PROMIS Sleep Disturbance and Sleep-Related Impairment scales, the Rosenberg Self-Esteem Scale, a self-report measure of physical appearance, and facial skin age estimated using an artificial intelligence–based application.

**Results:**

Compared with placebo, saffron resulted in greater reductions in DASS-21 depression scores (adjusted mean difference: 2.37; 95% CI: 0.23, 4.51; *d* = 0.48, *p* = 0.030). A clinically meaningful improvement in depressive symptoms (≥7-point reduction) was achieved by 48.8% of participants receiving saffron compared with 25.6% receiving placebo (*p* = 0.026). Saffron was also associated with greater improvements in self-esteem (adjusted mean difference: 1.43; 95% CI: 0.22, 2.64; *d* = 0.51; *p* = 0.022) and sleep-related impairment (adjusted mean difference: 2.99; 95% CI: 0.12, 5.86; *d* = 0.45; *p* = 0.041). No significant between-group differences were observed for sleep disturbance (*p* = 0.786), self-rated physical appearance (*p* = 0.964), or estimated facial skin age (*p* = 0.473). Saffron was well tolerated, with no serious adverse events reported.

**Conclusion:**

Supplementation with a saffron extract (Affron®) for 12 weeks was associated with improvements in depressive symptoms. Improvements were also observed in self-esteem and sleep-related impairment; however, these secondary findings should be considered exploratory and require confirmation in adequately powered studies. No significant group differences were observed for sleep disturbance, perceived physical appearance, or estimated facial skin age.

**Clinical trial registration:**

https://anzctr.org.au/ACTRN12625000638437p.aspx, ACTRN (registration number): ACTRN12625000638437p.

## 1 Introduction

Major depressive disorder affects approximately 5 to 10% of adults globally every year, with lifetime prevalence rates of 20% (1, 2). In Australian populations, 12-month and lifetime prevalence rates of 6 and 15%, respectively, have been reported, with higher rates in females than in males (3). Depression is associated with a reduced quality of life, increased rates of morbidity, and early mortality (2). In particular, depression has a significant comorbidity with insomnia and other sleep-related disturbances, where it is estimated that 40 to 75% of people with depression meet the criteria for clinical insomnia (4, 5). This is problematic as comorbid depression and insomnia increase the risk of treatment resistance and are associated with worsening general health (6). While sleep disturbances are included as a diagnostic criterion for depression, there exists a bidirectional relationship between sleep and depression (7).

As a treatment for depressive symptoms, saffron has undergone more than 20 clinical trials. Results from meta-analyses have confirmed that it can effectively reduce depressive symptoms in adults (8, 9). As a sleep-related aid, saffron has also undergone several clinical trials, which have confirmed it has sleep-enhancing effects (10–12). However, its effects on adults experiencing comorbid low mood and poor sleep have undergone limited investigation. In a recent study conducted in Australia, antidepressant effects were identified in adults with subclinical depression, and sleep-promoting benefits were identified, but only in a subset of participants experiencing more significant sleep disturbances (13).

In addition to the high comorbidity between depression and sleep, depressive symptoms are associated with reduced self-esteem, lower self-evaluations of physical appearance and skin quality, and increased skin aging (14–16). Moreover, poor sleepers report lower self-esteem (17), and inferior self-evaluations of physical appearance and skin quality (18, 19), which may be partially attributed to depressive symptoms (20). Insomnia and poor sleep are also associated with worsened objective measures of skin quality (21, 22).

Signs of facial aging, such as wrinkles and folds, poor skin tone and texture, and an imbalanced distribution of soft tissue, can have deleterious consequences on psychological and social wellbeing (23). In an examination of sex differences, it was shown that age-related changes in facial shape were similar in both sexes until around age 50, after which the female aging trajectory turned more sharply (24).

The effects of saffron on skin quality have received little attention. In an open-label study, there was some evidence of cosmetic effects when saffron, in conjunction with avocado oil, was applied topically (25). In a randomised-controlled study, when saffron (28 mg saffron extract standardised at 3.48% Crocin and 0.03% Safranal) was combined with several botanicals, its oral intake resulted in facial pigmentation (26). In another randomised-controlled study, improvements in a dermatological assessment of skin radiance after the oral administration of a nutraceutical supplement containing saffron were reported (27). In a cell-based study, skin protective effects were also demonstrated from a saffron extract on human dermal fibroblasts (28).

The primary objective of this study was to examine the effects of a saffron extract (Affron®) on depressive symptoms and sleep-related outcomes in women experiencing comorbid depressive symptoms and sleep disturbances. In addition, its influence on self-esteem, self-perceived physical appearance and skin quality, as well as objective measures of facial skin age using artificial intelligence (AI) technology, was also investigated. Women aged 50–70 years were selected because depressive symptoms, sleep disturbances, and age-related changes in facial appearance are particularly relevant during and after the menopausal transition, a period associated with increased vulnerability to mood disturbances, impaired sleep, and accelerated facial ageing (24, 29, 30). It was hypothesised that saffron would be associated with improvements in these parameters through the following mechanisms: (1) improvements in mood and sleep quality would result in generally more positive thoughts, leading to greater improvements in self-esteem and more positive physical self-evaluations; (2) depression and poor sleep are associated with increased oxidative stress and free radical activity (31, 32). As increased oxidative stress is associated with increased skin aging (33, 34), through its antioxidant effects, saffron may reduce skin aging; (3) tyrosinase inhibitors are used as a treatment for skin diseases and hyperpigmentation (35). Saffron can influence tyrosinase activity (28), thereby potentially influencing skin health; (4) chronic stress increases the risk of skin-related diseases and can affect skin barrier function, impair wound healing, and promote the release of pro-inflammatory cytokines, thereby influencing skin conditions (36). Through its anti-stress effects, saffron may affect dermatological health; and (5) elevated pro-inflammatory cytokine activation can contribute to skin conditions (37). Through its anti-inflammatory effects, saffron may influence skin health.

## 2 Materials and methods

### 2.1 Study design and procedures

Ethics approval was obtained from the National Institute of Integrative Medicine Human Research Ethics Committee (approval number 0155E_2025), and written informed consent was acquired from all participants at visit 1. This trial was prospectively registered with the Australian and New Zealand Clinical Trials Registry (ACTRN12625000638437p).

**Figure 1:**
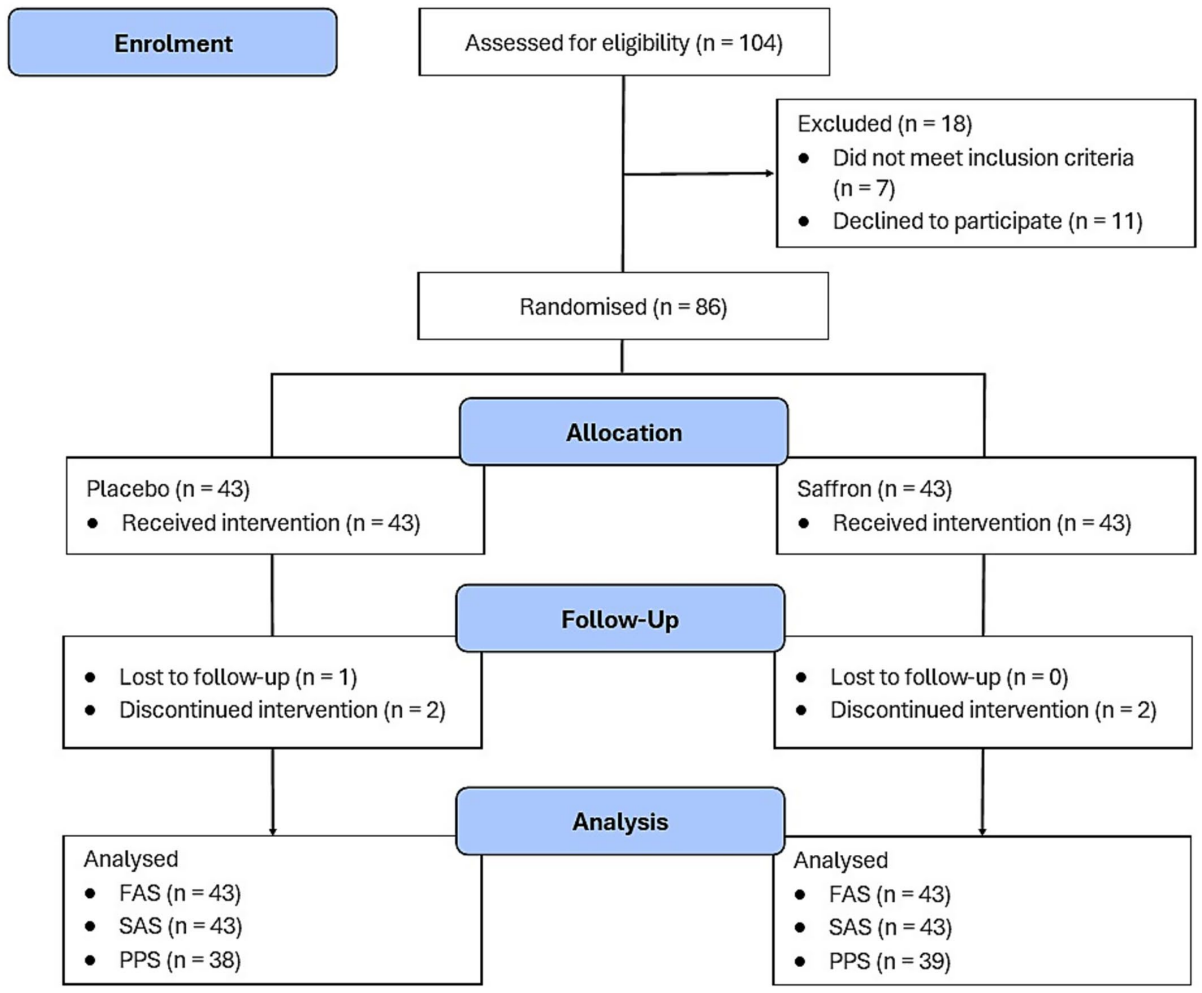
Systematic illustration of study design. FAS, full analysis set; PPS, per protocol set; SAS, safety analysis set.

This was a 12-week, two-arm, parallel-group, randomised, double-blind, placebo-controlled trial (Figure 1). Recruitment occurred between July and August 2025 through social media and emails to an in-house database. Applicants completed a screening survey that collected demographic details, health and medical status, and medication intake. Volunteers also completed the Depression, Anxiety, and Stress Scale, Depression Subscale (DASS-D) and the PROMIS Sleep Disturbance Scale. To proceed further, participants needed to score between 10 and 27 on the DASS-D (indicating mild-to-moderately-severe depressive symptoms) and to obtain a T-score of ≥ 56.5 on the PROMIS-Sleep Disturbance scale (indicating a score above the 75th percentile).

Applicants were then contacted by a researcher for a telephone interview. During this interview, a more comprehensive assessment of the eligibility criteria was undertaken, relevant demographic and sociographic details were obtained, and a full explanation of the study was provided. If eligible and willing to participate in the study, applicants attended an in-person assessment appointment, 3 to 10 working days later. At visit 1, the following tasks were undertaken: (1) completion of the informed consent form; (2) a researcher measured the participant’s weight, height, and seated blood pressure; (3) the participant completed self-report questionnaires; (4) a researcher took a photo of the participant’s face using a smartphone for an analysis of their skin age; and (5) the participant was given the study tablets. Participants completed the self-report questionnaires online at weeks 4 and 8, and when they returned at week 12 for visit 2, where similar assessments to visit 1 were undertaken.

### 2.2 Randomisation and blinding

Participants were blinded throughout the study, and the researchers and statistician were blinded to the treatment allocation until all outcomes were collected and a blind review was completed. Participants were randomly allocated to one of two groups (saffron or placebo) on a 1:1 basis using a randomisation calculator with a randomisation structure of eight permuted blocks, with 10 participants per block. A participant identification number was assigned in the order of participant enrolment. The randomisation sequence was generated by the study sponsor, who was not directly involved in volunteer recruitment. All tablets were packed in identical containers, and the study sponsor held bottle group-allocation codes.

### 2.3 Participants

#### 2.3.1 Inclusion criteria

Inclusion criteria for the trial comprised the following: Females aged between 50 and 70 years; currently experiencing low mood as demonstrated by a score of 10 to 27 on the DASS-D; a score greater than the 75th percentile (T-score ≥ 56.5) on the PROMIS Sleep Disturbance scale; non-smoker; had a body mass index (BMI) between 18 and 35 kg/m^2^; and had no plan to commence new treatments for mood, sleep, or skin quality over the study period.

#### 2.3.2 Exclusion criteria

The exclusion criteria comprised the following: was currently receiving regular psychological therapy; had a recently diagnosed or unmanaged medical condition, including but not limited to diabetes, hypertension, cardiovascular disease, autoimmune disease, endocrine disease, or cancer; had a diagnosis of a psychiatric disorder (other than mild-to-moderate depression and/or anxiety); had a neurological condition, including but not limited to Parkinson’s or Alzheimer’s disease; regularly took medications, including but not limited to anticonvulsants, benzodiazepines, opioids, corticosteroids, or immunosuppressants; changed medication in the last 3 months or planned to change during the study period; in the previous 3 months, commenced or changed the dose of nutritional and/or herbal supplements that may impact treatment outcome; was currently taking supplements containing saffron; consumed more than 14 standard drinks of alcohol per week; had a current or 12-month history of regular illicit drug use; had a planned major lifestyle change in the next 3 months; was pregnant, breastfeeding, or intended to become pregnant during the study period; had any significant surgeries that have continued to affect daily function over the last year; participated in any other clinical trial in the previous month.

### 2.4 Interventions

The intervention comprised either a saffron extract (Affron®) or a placebo (microcrystalline cellulose). Participants were instructed to take one tablet in the morning and one in the evening, with or without food, for 12 weeks. Each active tablet contained 14 mg of Affron®, delivering a daily dose of 28 mg. The active and placebo tablets were identical, matched in shape, colour, and size. Both tablets contained the same excipients comprising microcrystalline cellulose, calcium phosphate dihydrate, magnesium stearate, and silicon dioxide. Affron® is produced from the stigmas of *Crocus sativus* L. and standardised to contain >3.5% Lepticrosalides® (3.48% crocins + 0.03% safranal), a measure of bioactive compounds contained in saffron, including safranal and crocin isomers. Further details about Affron® are included in the Supplementary file. A researcher-administered pill count assessed adherence to tablet intake at visit 2. Treatment blinding was evaluated by asking participants to guess their group allocation (saffron, placebo, or unsure) at the end of the study, along with a reason for their prediction.

### 2.5 Outcome measures

#### 2.5.1 Primary outcome measure

##### 2.5.1.1 Depression, Anxiety, and Stress Scale Depression (DASS-D) score

The DASS-21 is a validated self-report questionnaire that assesses symptoms associated with depression, anxiety, and stress over the last 7 days (38). Since an examination of changes in depression/ low mood was the primary objective of this study, only the depression subscale (DASS-D), which comprises 7 items, assessing depressive symptoms, was administered. The DASS-D score was selected, *a priori*, as the primary outcome measure. In a population of adult outpatients with depression, a reduction of 6.15 or more was determined as the clinically significant change index (39). Therefore, a reduction of ≥7 points on the DASS-D was considered clinically significant.

#### 2.5.2 Secondary outcome measures

##### 2.5.2.1 PROMIS Sleep Disturbance and Sleep-Related Impairment Scale (PROMIS Sleep)

The PROMIS Sleep is a 16-item self-report inventory that measures sleep quality and sleep-related impairment over the last week. Questions are rated using a 5-point Likert scale, where component scores are calculated for sleep disturbance and sleep-related impairment (40).

##### 2.5.2.2 Rosenberg Self-Esteem Questionnaire (RSE)

The RSE is a validated 10-item self-report questionnaire that measures global self-worth by measuring both positive and negative feelings about the self (41). All items are answered using a 4-point Likert scale ranging from strongly agree (4) to strongly disagree (1).

##### 2.5.2.3 Satisfaction with attractiveness, skin health and general appearance questionnaire (SASA)

The SASA is a 6-item questionnaire that has previously been used in a study examining the effects of sleep on skin ageing, whereby good sleepers reported a significantly better perception of their appearance and physical attractiveness compared with poor sleepers (21). Using a 5-point Likert scale (poor to excellent), participants rated their physical appearance.

##### 2.5.2.4 Patient Global Impression of Change (PGIC)

The PGIC is a measure of a person’s belief about the efficacy of treatment (42). Respondents are asked to estimate the difference between their current and previous health state based on a Likert scale ranging from (1) very much improved to (7) very much worse.

##### 2.5.2.5 SkinFace (Topazium Artificial Intelligence S. L., Madrid)

SkinFace was selected as an objective and non-invasive measure of facial skin age. The application uses machine-learning algorithms trained on a large image dataset to estimate facial age from standardised facial photographs. Although AI-based facial age estimation tools continue to be validated, they offer a practical approach for objectively assessing global facial ageing characteristics in clinical and community-based studies where comprehensive dermatological imaging systems may not be feasible.

#### 2.5.3 Safety measures

Tolerance to tablet intake was assessed at weeks 4 and 8 using an online questionnaire enquiring about the experience of any adverse events. Moreover, during visit 2, researchers questioned participants about any adverse events. At visit 2, participants also completed the Patient Global Assessment of Tolerability to Therapy (PGATT), in which they rated their tolerance to tablet intake on a scale from poor to excellent.

### 2.6 Sample size calculations

An *a priori* power analysis was undertaken to estimate the required sample size (based on a single outcome variable). To estimate the effect size, data from a study we completed in 2024 on 200 adults was used (13). In a subset of 40 women aged over 50 years experiencing sleep difficulties, a Cohen’s d effect size of 0.6 was identified for changes in the DASS-D score. Based on this effect size, a power of 80%, and a type one error rate (alpha) of 5%, the total number of participants required to find an effect is 72. Assuming a 10% dropout rate, a sample size of 80 people was predicted to find an effect on the primary outcome measure.

### 2.7 Statistical analysis

Outcome analyses for the self-report questionnaires were conducted on the full analysis set (FAS) using an intention-to-treat analysis, and on the per-protocol set (PPS). FAS represents the subset of participants who were randomised, consumed at least one dose of the investigational tablets, and had available efficacy data. PPS was defined as the subset of participants who were randomised, who consumed at least one dose of the trial tablets, had available efficacy data, and had no major protocol deviations (e.g., withdrew from the study, consumed less than 80% of tablets, started prohibited concomitant medications, had missing data, and/or completed assessments outside proposed time windows).

A univariate ANOVA assessed differences between intervention groups (placebo and saffron) for treatment outcomes comprising the DASS-D, RSE, SASA, and PROMIS Sleep scores. Changes in scores from day 0 to week 12 were examined between the two intervention groups, with age, BMI, and their corresponding baseline values as covariates. Estimated means and standard errors are included in the relevant tables. Cohen’s *d* effect sizes were calculated for each outcome measure.

As skin age data were not normally distributed and transformations could not normalise the data, a non-parametric test (independent-samples Mann–Whitney *U*-test) was used to compare group differences in changes in skin age over time.

To examine within-group changes over time, a repeated-measures ANOVA was used with the time points day 0, weeks 4, 8, and 12 included. The covariates age and BMI were included. Estimated means and standard errors are included in the relevant tables. Group differences in PGIC and PGATT ratings were analysed using a chi-square test. All data were analysed using SPSS (version 31; IBM, Armonk, NY) and the critical *p*-value was set at *p* ≤ 0.05 (two-sided) for all analyses. As there was only one *a priori* primary outcome measure, no adjustment for multiple testing was made to the *p*-value. Secondary outcomes were exploratory and hypothesis-generating. Therefore, no multiplicity adjustment was applied.

## 3 Results

### 3.1 Study population

A total of 218 people completed the online screening questionnaire, 104 underwent telephone screening, and 86 were randomised (80 participants were originally planned for recruitment). Of the 104 people interviewed, the most common reason for exclusion was withdrawal of consent after the telephone interview (*n* = 11). Baseline sociodemographic and clinical characteristics are detailed in Table 1.

**Table 1:** Baseline sociodemographic and clinical characteristics.

Sociodemographic and clinical characteristics		Placebo (*n* = 43)	Saffron (*n* = 43)
Age (years)	Mean	62.24	63.06
	SE	0.83	0.85
Height (m)	Mean	1.64	1.64
	SE	0.01	0.01
Weight (kg)	Mean	72.66	72.04
	SE	1.34	2.06
BMI (kg/m^2^)	Mean	27.00	26.68
	SE	0.50	0.67
Systolic blood pressure (mmHg)	Mean	127.30	125.58
	SE	2.42	2.52
Diastolic blood pressure (mmHg)	Mean	78.28	78.26
	SE	1.44	1.41
Marital status (*n*)	Single	16	19
	Married/de facto	27	24
Menopausal status	Perimenopause	4	3
	Post-menopause	39	40
Education (*n*)	Secondary	25	20
	Tertiary	10	15
	Post-graduate	8	8
Exercise days per week	Mean	1.51	1.33
	SE	0.26	0.30
Occupation (*n*)	Retired	16	21
	Professional	6	6
	Technicians and associated trades	6	5
	Elementary occupation	4	3
	Clerical support worker	3	2
	Services and sales worker	2	2
	Manager	2	2
	Unemployed	2	1
	Craft and related trades worker	2	1
DASS-D score (screening)	Mean	16.60	16.23
	SE	0.73	0.87
DASS-D Score (Day 0)	Mean	11.81	13.30
	SE	0.85	0.92
RSE total score	Mean	27.81	27.77
	SE	0.70	0.74
SASA score	Mean	16.47	16.02
	SE	0.79	0.65
Skin age (yrs)	Mean	57.93	59.33
	SE	1.39	1.15
PROMIS Sleep: sleep disturbance (T-score) (screening)	Mean	63.74	61.97
	SE	0.72	0.59
PROMIS Sleep: sleep disturbance (T-score)	Mean	61.46	59.86
	SE	0.82	0.89
PROMIS Sleep: sleep-related impairment (T-score)	Mean	56.62	57.41
	SE	1.17	0.71

BMI, Body Mass Index; DASS-D, Depression, Anxiety, and Stress Scale 21: Depression Scale; PROMIS Sleep, PROMIS Sleep Disturbance and Sleep-Related Impairment Scale; RSE, Rosenberg Self-Esteem Questionnaire; SASA, Satisfaction with attractiveness, skin health and general appearance questionnaire; SE, standard error.

### 3.2 Outcome measures

#### 3.2.1 DASS-D

**Figure 2:**
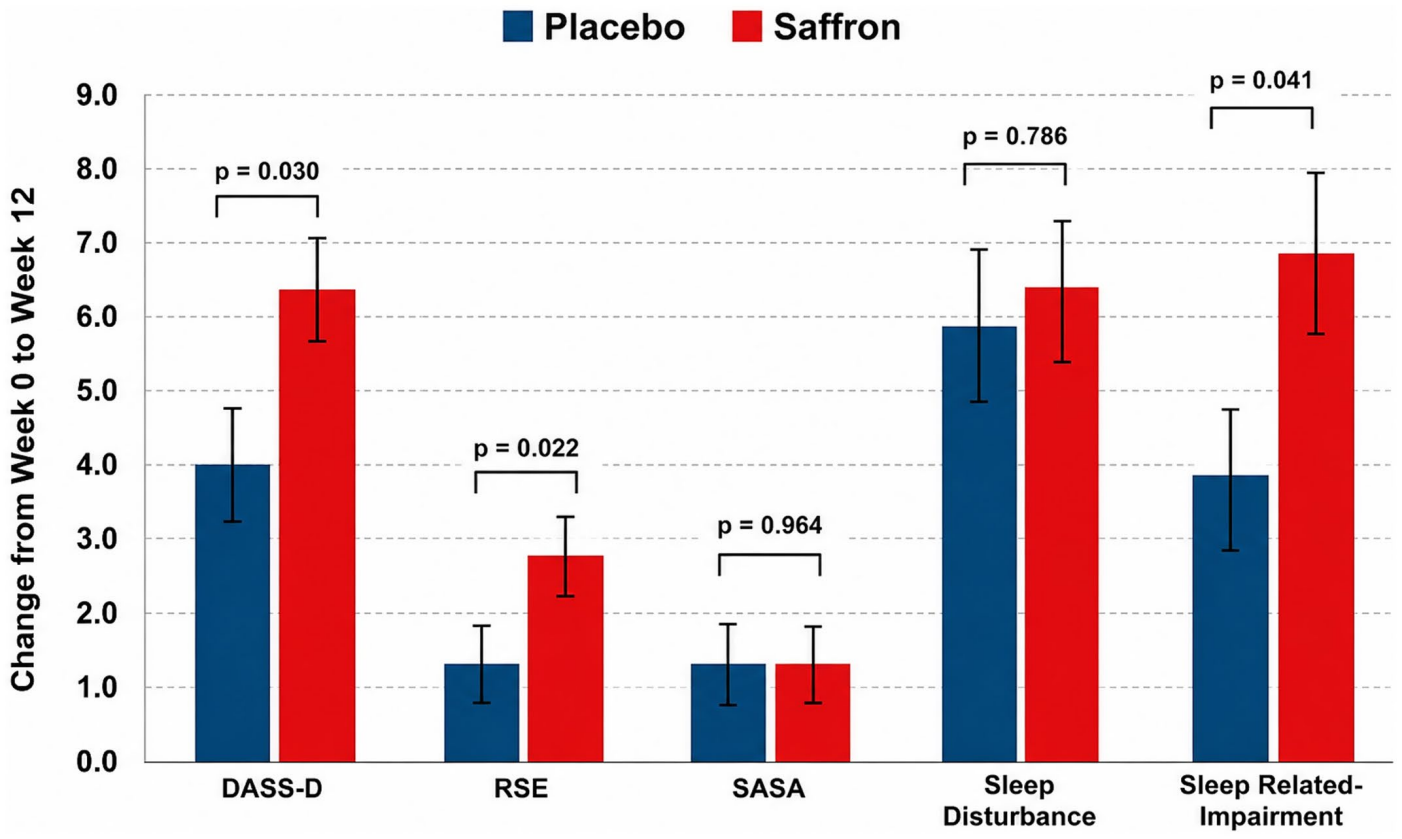
Change in self-report scores from weeks 0 to 12 (vertical bars represent standard error) (FAS). DASS-D, Depression, Anxiety, and Stress Scale 21: Depression Scale; FAS, full analysis set; RSE, Rosenberg Self-Esteem Questionnaire; SASA, Satisfaction with attractiveness, skin health and general appearance questionnaire.

As demonstrated in Table 2 and Figure 2, there was a statistically significant group difference in the change in the DASS-D score from day 0 to week 12 (adjusted mean difference: 2.37; 95% CI: 0.23, 4.51; *d* = 0.48, *p* = 0.030). From day 0 to week 12, in the saffron group, the mean score decreased by 6.4 points (95% CI: 4.87, 7.87, *p* < 0.001) and in the placebo group, it decreased by 4.0 points (95% CI: 2.50, 5.50, *p* < 0.001). After controlling for baseline values (mean = 12.56), the DASS-D score decreased by 50.7% in the saffron group, while in the placebo group it decreased by 31.9%. An analysis of the PPS revealed similar findings (Supplementary Table 1).

**Table 2:** Change in outcome measures from Week 0 to Week 12 (estimated marginal means) (FAS).

Outcome measures		Placebo (*n* = 43)						Saffron (*n* = 43)						*p*-value^b^	Cohen’s *d*
		Week 0^a^	Week 4^a^	Week 8^a^	Week 12^a^	Change^b^	*p*-value^a^	Week 0^a^	Week 4^a^	Week 8^a^	Week 12^a^	Change^b^	*p*-value^a^		
DASS-D score	Mean	11.71	9.82	8.27	8.00	4.00	<0.001	13.40	9.02	7.57	6.74	6.37	<0.001	0.030	0.48
	SE	0.88	1.03	0.97	0.94	0.75		0.88	1.03	0.97	0.94	0.75			
RSE score	Mean	27.69	29.08	30.07	30.47	1.34	0.019	27.89	28.62	28.98	29.22	2.77	<0.001	0.022	0.51
	SE	0.71	0.76	0.74	0.75	0.43		0.71	0.76	0.74	0.75	0.43			
SASA score	Mean	16.51	16.74	17.42	17.79	1.29	0.036	15.98	16.63	17.44	17.27	1.27	0.033	0.964	0.01
	SE	0.73	0.78	0.74	0.80	0.45		0.73	0.78	0.74	0.80	0.45			
PROMIS Sleep Disturbance (T-score)	Mean	61.44	56.21	55.91	55.08	5.97	<0.001	59.88	54.10	53.36	53.93	6.35	<0.001	0.786	0.06
	SE	0.86	0.97	1.06	1.06	0.98		0.86	0.97	1.06	1.06	0.98			
PROMIS Sleep-Related Impairment (T-score)	Mean	56.54	52.77	52.52	52.86	3.86	0.006	57.48	51.80	50.82	50.44	6.86	<0.001	0.041	0.45
	SE	0.97	1.11	1.21	1.17	1.02		0.97	1.11	1.21	1.17	1.02			
Skin age (yrs)	Mean	58.41	-	-	58.13	−0.43	0.812	58.84	-	-	57.87	−0.83	0.417	0.473^c^	NA
	SE	0.90	-	-	1.08	1.04		0.90	-	-	1.08	1.04			

^a^ *p*-values (within group) and estimated means are generated from repeated-measures ANOVAs adjusted for age and BMI.

^b^ *p*-values and estimated means (change from W0 to W12) generated from univariate ANOVAs adjusted for age, BMI, and corresponding baseline value.

^c^ *p*-value generated from an independent-samples Mann–Whitney *U*-test.

A greater proportion of participants in the saffron group achieved a clinically significant change in the DASS-D (≥7 points) than in the placebo group (*p* = 0.026). In the saffron group, 48.8% (*n* = 21) of participants achieved a clinically significant change, compared with 25.6% (*n* = 11) in the placebo group.

#### 3.2.2 RSE Score

As demonstrated in Table 2 and Figure 2, there was a statistically significant group difference in the change in the RSE score from day 0 to week 12 (adjusted mean difference: 1.43; 95% CI: 0.22, 2.64; *d* = 0.51, *p* = 0.022). From day 0 to week 12, in the saffron group, the mean score increased by 2.77 points (95% CI: 1.92, 3.63, *p* < 0.001), whereas in the placebo group it increased by 1.34 points (95% CI: 0.50, 2.20, *p* = 0.019). After controlling for baseline scores (27.79), this represents a 10.0% improvement in the saffron group compared with a 4.8% improvement in the placebo group. An analysis of the PPS revealed similar findings (Supplementary Table 1).

#### 3.2.3 SASA Score

As demonstrated in Table 2 and Figure 2, there was no statistically significant group difference in the change in scores from day 0 to week 12 (adjusted mean difference: 0.029; 95% CI: −1.25, 1.31; *d* = 0.01, *p* = 0.964). An analysis of the PPS revealed similar findings (Supplementary Table 1).

#### 3.2.4 PROMIS Sleep Disturbance Score

As demonstrated in Table 2 and Figure 2, there was no statistically significant group difference in the change in the PROMIS Sleep Disturbance T-score from day 0 to week 12 (adjusted mean difference: 0.38; 95% CI: −2.40, 3.16; *d* = 0.059, *p* = 0.786).

#### 3.2.5 PROMIS Sleep-Related Impairment Score

As demonstrated in Table 2 and Figure 2, there was a statistically significant group difference in the change in the PROMIS Sleep-Related Impairment T-score from day 0 to week 12 (adjusted mean difference: 2.99; 95% CI: 0.12, 5.86; *d* = 0.45, *p* = 0.041). From day 0 to week 12, in the saffron group, the mean score decreased by 6.86 points (95% CI: 4.83, 8.88, *p* < 0.001), whereas in the placebo group it decreased by 3.86 points (95% CI: 1.84, 5.89, *p* = 0.006). An analysis of the PPS revealed similar findings (Supplementary Table 1).

#### 3.2.6 PGIC ratings

There were no group differences in PGIC ratings (*p* = 0.630) (Supplementary Table 2). In the saffron group, 19 participants (46.3%) reported mood improvement, compared with 17 (42.5%) in the placebo group.

#### 3.2.7 Skin age

As detailed in Table 2 and Supplementary Figure 1, there was no statistically significant group difference in the change in skin age from day 0 to week 12 (*p* = 0.473).

### 3.3 Intake of supplements

Study bottles with remaining tablets were returned by 77 (95%) of participants at visit 2. Based on a tablet count conducted by a researcher, only one person took less than 80% of their study tablets (58% compliance). Participants who did not return their IP (*n* = 4) reported consistently taking their study tablets, although a tablet count could not confirm this.

### 3.4 Adverse reactions and treatment discontinuation

Participants reported no serious AEs, and the frequency of AEs classified as possibly or probably related to tablet intake was similar (Supplementary Table 3). In the saffron group, 41 (95.3%) participants did not report an adverse reaction compared to 40 (93.0%) in the placebo group. The PGATT ratings completed on week 12 demonstrated that in the saffron group, 100% of participants reported good or excellent tolerability to the tablets, compared with 97.6% in the placebo group. Moderate tolerability to the IP was reported by 1 participant (2.4%) in the placebo group (Supplementary Table 4).

### 3.5 Efficacy of participant blinding

To assess the effectiveness of blinding during the trial, participants were asked to predict their condition allocation (i.e., placebo, saffron, or unsure) at the end of the study. In the saffron group, only 12.5% of participants correctly predicted treatment allocation, and only one participant cited unblinding due to the tablets’ aroma. In the placebo group, 42.5% of participants correctly guessed treatment allocation; however, the primary reason cited for the correct prediction was the absence of noticeable treatment effects. No participants in the placebo group reported unblinding due to the IP’s taste, appearance, or aroma.

## 4 Discussion

The aims of the study were to investigate the effects of 12 weeks of supplementation with a saffron extract (Affron®) on self-reported depressive symptoms, sleep quality, self-esteem, physical appearance, and skin age in women aged 50 to 70 years experiencing low mood and poor sleep. Based on the primary outcome measure (DASS-D), Affron® resulted in an adjusted mean difference of 2.37 points relative to placebo (95% CI: 0.23, 4.51), corresponding to a moderate effect size (*d* = 0.48). Importantly, the clinical relevance of these findings is supported by the responder analysis. Nearly half of participants receiving saffron (48.8%) achieved a clinically significant reduction in depressive symptoms, defined *a priori* as a reduction of ≥7 points on the DASS-D, compared with approximately one-quarter of participants receiving placebo (25.6%). Based on these responder rates, the estimated number needed to treat was approximately 4.3, indicating that treatment of approximately four to five women with saffron would result in one additional clinically significant improvement in depressive symptoms beyond that observed with placebo. Together, these findings suggest that clinically meaningful improvements occurred in a subset of participants despite the substantial placebo response commonly observed in depression trials. These results are consistent with previous studies of Affron®, in which improvements in depressive symptoms were identified in adults with subclinical depression (13), perimenopausal women (43), and healthy adults with low mood (44). Nevertheless, replication in larger studies is required to further establish the magnitude and consistency of these effects.

An improvement in the PROMIS Sleep-Related Impairment score was observed in this study, with a Cohen’s d effect size of 0.45. However, no significant between-group difference was identified on the PROMIS Sleep Disturbance scale, which serves as the primary measure of perceived sleep quality. Beneficial effects on sleep outcomes have been identified in previous Affron® trials (13, 45, 46), which utilised various self-report sleep measures. In a recent trial, improvements in sleep disturbance, but not in sleep-related impairment, were observed in a subset of participants with sleep-related problems, using the same sleep measure employed in the current study (PROMIS Sleep). Therefore, the findings differ from those reported in the previous trial. This is likely due to the large placebo response in the current study’s sleep disturbance score. Placebo responses are common in mood and sleep-related trials (47–49), which can affect the ability of randomised controlled trials to detect group differences. Utilising outcome measures that are less influenced by placebo responses, such as objective outcome sleep measures (49), biological measures, and tools that may more sensitively detect changes, will be important. It is essential to note that the greater improvements in sleep-related impairment observed in the Affron® group may also be partly due to mood-related improvements among participants. The PROMIS Sleep-Related Impairment subscale includes questions around alertness, tiredness, irritability, and concentration, which are also symptoms of low mood and often improve following the alleviation of depressive symptoms.

A novel exploratory finding from this study was the improvement in self-esteem observed among participants supplemented with saffron. This has not been previously examined, and the results of this study demonstrated that, along with improvements in mood and sleep, saffron supplementation may also result in a modest enhancement in self-esteem (i.e., a person’s evaluation of their worth as an individual). However, given that self-esteem was a secondary outcome and the study was not powered specifically for this endpoint, these findings should be interpreted cautiously until replicated in larger trials. As there were no group differences in changes in self-rated physical appearance or in skin-age measures, it is possible that improvements in self-esteem were due to mood-related improvements. Research confirms that there is a bidirectional relationship between depression and self-esteem (50). However, it is important to note that a reduction in depressive symptoms does not automatically lead to improvements in self-esteem. Haehner et al., (15) reported that although there is a strong correlation between depression and self-esteem, these are different constructs with differing causative influences and patterns of change over time. In particular, self-esteem and depression can be influenced by different adverse events; low self-esteem is not a defining feature of depression; and self-esteem and depressive symptoms differ in their development and stability over time, with self-esteem being more stable. Improvements in self-esteem from antidepressant medications have undergone limited investigation. However, in a meta-analysis on youths with depression, it was concluded that antidepressants yielded no significant advantage over placebo in self-esteem (51). From a physiological mechanistic perspective, a relationship between self-esteem, cortisol (52–54), serotonin transporter gene polymorphism (55), and hippocampal activity (56, 57) has been identified. Moreover, increased inflammation induced by a low-dose endotoxin has been shown to reduce body-related self-esteem (58), and interleukin-6 concentrations were negatively correlated with self-esteem scores in metabolically unhealthy people with obesity (59). Although correlation does not confirm causation, saffron may influence self-esteem through these biological mechanisms, as it has been shown to have anti-inflammatory, neurotransmitter activity-enhancing, and cortisol-lowering effects (60, 61), and can influence hippocampal activity (62, 63).

### 4.1 Strengths, limitations and directions for future research

This study adds to the body of evidence of the antidepressant efficacy of saffron, which has now been extended to women aged 50 to 70 with comorbid low mood and poor sleep. However, the present study was designed to evaluate the efficacy of saffron relative to placebo and was not intended to compare saffron with established treatments such as antidepressant medications or psychological therapies. Accordingly, the findings should not be interpreted as evidence of comparative efficacy. Instead, saffron may be considered a potential adjunctive or alternative intervention for women experiencing mild-to-moderate depressive symptoms and sleep disturbances who are unwilling or unable to access conventional treatments. Moreover, the findings relating to secondary outcomes, including self-esteem and sleep-related impairment, should be interpreted as exploratory and hypothesis-generating and require replication in future studies. The study was specifically powered to detect changes in the primary outcome measure (DASS-D) and was not powered to detect between-group differences in secondary or exploratory outcomes, such as self-esteem, self-perceived appearance, or AI-derived skin age. Consequently, the absence of significant effects for appearance-related outcomes and the positive findings for self-esteem should be interpreted cautiously until replicated in adequately powered studies specifically designed to evaluate these outcomes. As no multiplicity adjustment was applied, there is also an increased risk of type I error for secondary outcomes, further supporting the need for independent confirmation. It is also important to note that these findings are also specific to women aged 50–70 years experiencing low mood and poor sleep and should not be generalised to men, younger adults, or individuals with major depressive disorder.

As an exploratory examination, the effects of saffron on skin health were investigated. However, limitations in the outcome measure used to assess skin health compromise the robustness of the findings. SkinFace, which utilises participants’ facial photos, should be regarded as an exploratory assessment tool. Although promising, further validation is required before it can be considered a robust clinical endpoint. The inclusion of SkinFace was intended to provide an objective complement to self-reported appearance assessments. Traditional dermatological assessments often require specialised imaging equipment, expert clinical raters, or invasive procedures, which were not feasible within the current study’s design. AI-based facial age estimation tools offer a practical and standardised method for evaluating overall facial ageing characteristics. However, such technologies should currently be regarded as exploratory research tools, and further validation against established dermatological measures is required. Wrinkles, folds, changes in tone or skin texture, or other specific facial signs related to ageing were not investigated in this study. Therefore, to provide more robust findings on the effects of saffron on skin health, objective and validated measures should be utilised. This includes a combination of biophysical, imaging, and questionnaire-based tools that objectively quantify parameters like hydration, barrier function, elasticity, and texture (64).

The improvements in self-esteem identified in the study are a novel finding, as changes in self-esteem are not regularly measured in antidepressant studies. However, it is worth noting that improvements in the RSE score were modest, with a 10% increase in the saffron group compared to a 4.8% increase in the placebo group. Although modest, compared to depression measures, self-esteem is a more stable construct with test–retest correlations of 0.85 and 0.75 at 2-week and 6-month intervals, respectively (65). Moreover, longitudinal studies show that self-esteem typically peaks around age 60 years (66); therefore, limiting potential changes that may occur over a short 12-week intervention in an age cohort of 50 to 70-year-olds, where self-esteem may already be at its peak. Further research in this area is warranted across different populations, age cohorts, and longer time intervals, particularly as clinically meaningful scores have not been identified.

While there were no group differences in changes in SASA scores, it is important to note that baseline scores were approximately 16. This suggests a generally satisfied perception of physical attractiveness at baseline. Moreover, baseline scores were similar to those reported in a previous study using the SASA in healthy, non-depressed women aged 30 to 50 years experiencing poor sleep (21). Therefore, to evaluate the effects of saffron on self-perceived physical attractiveness, it would be prudent in future trials to recruit women with low perceptions of physical attractiveness.

Previous antidepressant and sleep studies on saffron have primarily delivered daily doses ranging from 28 to 30 mg for 4 to 12 weeks (12, 67). In relation to Affron®, the saffron extract used in this study, dosing has primarily comprised 14 mg twice daily for 4 to 12 weeks (13, 43, 46). While mood and sleep-enhancing efficacy have been demonstrated, further research utilising larger sample sizes, longer treatment periods, and varying dosing regimens will be important to help establish optimal treatment guidelines. Moreover, dose-escalation studies for partial or non-responders have not been previously investigated and efficacy compared to commonly used psychological therapy or antidepressant medications has not been undertaken in this cohort of women aged 50 to 70 years. For changes in skin health, higher doses may also be necessary. However, cosmetic benefits were reported in a study using a saffron extract with a similar standardisation and dose as used in the current study, albeit administered orally with other botanicals (26).

It is important to note that the findings of the present study are specific to the saffron extract administered, Affron®. This extract is standardised to contain >3.5% Lepticrosalides®, a patented measure of its bioactive constituents, comprising predominantly crocins (3.48%) and smaller amounts of safranal (0.03%). Accordingly, the clinical effects observed in this study are likely attributable primarily to the crocin fraction, with only a limited contribution from the volatile safranal component. Whether comparable clinical efficacy and tolerability can be achieved with saffron extracts of different phytochemical profiles, or with isolated constituents such as crocins or safranal alone, warrants further investigation.

Although the aqueous dispersibility of Affron® has not been extensively characterised directly, its principal bioactive compounds, particularly crocins, are hydrophilic glycosylated carotenoids with well-established water-soluble properties (68, 69). It should also be noted that colourimetric parameters, including a*, b*, and Yellow Index values, were not assessed during the analytical characterisation of the extract used in this current study. Instead, the phytochemical analyses focused primarily on crocin composition and related saffron constituents. Crocins are responsible for the characteristic colouring properties and colouring strength of saffron extracts (70). However, the inclusion of colourimetric analyses may have provided additional information regarding pigment characteristics and batch standardisation. Future studies should consider incorporating such assessments. Finally, the saffron extract used in this trial was supplied with a manufacturer-issued Certificate of Analysis detailing standardisation, purity, and contaminant testing. However, independent analytical verification was not undertaken, which should be acknowledged as a limitation of the study.

While this study identified an improvement in sleep-related impairment, no significant between-group difference was observed on the PROMIS Sleep Disturbance scale. Accordingly, these findings should not be interpreted as definitive evidence of an overall sleep-promoting effect and should be confirmed using objective sleep measures and adequately powered trials. Given the substantial placebo responses commonly observed in sleep-related trials, the inclusion of objective sleep measures such as actigraphy or polysomnography will be important in future studies to help substantiate treatment effects and provide a more comprehensive assessment of sleep-related outcomes.

The biological mechanisms underlying the antidepressant and sleep-promoting effects of saffron also require further investigation. Several mechanisms have been proposed, including modulation of neurotransmitter activity, anti-inflammatory and antioxidant effects, regulation of the hypothalamic–pituitary–adrenal axis, and neuroprotective actions (60). Future mechanistic studies incorporating biological and physiological outcome measures may help clarify the relative contribution of these pathways.

Finally, although the study achieved its recruitment target and was adequately powered for the primary outcome, the sample size was modest and may limit the precision and reproducibility of the observed effect estimates. Larger, adequately powered multicentre trials will be important to confirm the magnitude, consistency, and generalisability of these findings.

### 4.2 Conclusion

In summary, this study provides evidence that supplementation with a saffron extract (Affron®) for 12 weeks was associated with improvements in depressive symptoms in women aged 50 to 70 years experiencing low mood and poor sleep. Improvements in self-esteem and sleep-related impairment were also observed; however, these secondary findings should be regarded as exploratory and hypothesis-generating, as the study was powered only for the primary outcome. Moreover, no significant between-group difference was observed on the PROMIS Sleep Disturbance scale, indicating that evidence for sleep-specific efficacy remains preliminary. Confirmation in larger, adequately powered studies is warranted. No significant effects were identified on self-perceived appearance or AI-derived measures of skin age. Future trials incorporating larger sample sizes, alternative dosing regimens, and validated objective measures of sleep and skin health will help establish the consistency, clinical relevance, and generalisability of these findings.

## Data Availability

The raw data supporting the conclusions of this article will be made available by the authors, without undue reservation.
