# Supplementary material for: The effects of a saffron extract (Affron®) on mood, sleep, self-esteem, and exploratory measures of physical appearance in women aged 50 to 70 years experiencing low mood and poor sleep: a randomised, double-blind, placebo-controlled trial
Source: Front Nutr. 2026 Jun 17;13:1838513. doi: 10.3389/fnut.2026.1838513 (PMC13318664; doi:10.3389/fnut.2026.1838513)
Supplement: Supplementary file 1 [file Data_Sheet_1.pdf]

Supplementary Material

The effects of a saffron extract (Affron®) on mood, sleep, and physical appearance attributes in women aged 50 to 70 years experiencing low mood and poor sleep: a randomised, double-blind, placebo-controlled trial

Author names:

[1,2] Adrian L Lopresti - ORCID iD: 0000-0002-6409-7839

[1] Stephen J Smith - ORCID iD: 0000-0002-3875-4815

Author affiliations:

[1]Clinical Research Australia, Perth, Western Australia, 6023, Australia

[2]College of Science, Health, Engineering and Education, Murdoch University, Perth, Western Australia, 6150, Australia

Corresponding author: Adrian Lopresti, Clinical Research Australia, 38 Arnisdale Road Duncraig, Western Australia 6023, adrian@clinicalresearch.com.au

Details on the Saffron Extract (Affron®) used in this study

Affron® batch coded SAF-30695-PH was manufactured in December 2023. The batch was analysed, and a Certificate of Analysis was issued before the investigational product was used in this trial.

Lepticrosalides® is a registered trademark of Pharmactive and refers to the sum of the concentration of active compounds (specifically total crocins and safranal) measured by High-Performance Liquid Chromatography (HPLC). Lepticrosalides® is a patented measure of bioactive compounds present in Affron®, with a minimum amount of 0.03% safranal and more than 3.48% crocins (dry basis) measured by HPLC, as reported in the patents WO 2017/182688 and ES 2573542B1 (Inarejos-Garcia et al., 2017; Inarejos-Garcia et al., 2016).

The quantification of active compounds was undertaken using the HPLC-DAD method published by Caballero-Ortega et al. (2007) and Mena-García et al (2023), as well as the procedures described in Pharmactive's cited patents, which are the standard procedures in Pharmactive's QC.

As detailed below, the Affron® batch complied with the typical crocin fingerprint of major crocins, as well as the minimum amount of crocins and safranal.

Table. Identification and characterisation of the Affron® extract batch

Batch ID and Manufacturing date		SAF-30695-PH. Manufactured in December 2023	
Chemical characterisation			
Analytical Method: HPLC-DAD according to Carmona et al. (2006)			
Crocins	Concentration (%)	Percentage of total crocins (%)	Patented range
trans-4-GG	2.13%	51%	45-65% of total crocins
trans-3-Gg	0.80%	19%	15-30% of total crocins
Total crocins	4.14%	100%	>3.48%
Safranal	0.049%	-	>0.03%
Picrocrocin	3.67%	-	>2.5%
Kaempferol derivatives	0.28%	-	-

Crocin nomenclature was adopted according to the classification proposed by Carmona et al. (2006) and subsequently used by Mena-Garcia et al. (2023). This nomenclature system is more widely employed in saffron phytochemical research than alternative classifications such as crocin I and II, which are more commonly associated with Gardenia-derived crocins. Within this framework, crocin I corresponds to trans-crocin 4 and crocin II to trans-crocin 3.

Figure. Chromatographic profile of Affron® SAF-30695-PH. A) Crocins (λ= 440 nm); B) Safranal (λ= 310 nm); C) Picrocrocin (λ= 250 nm).

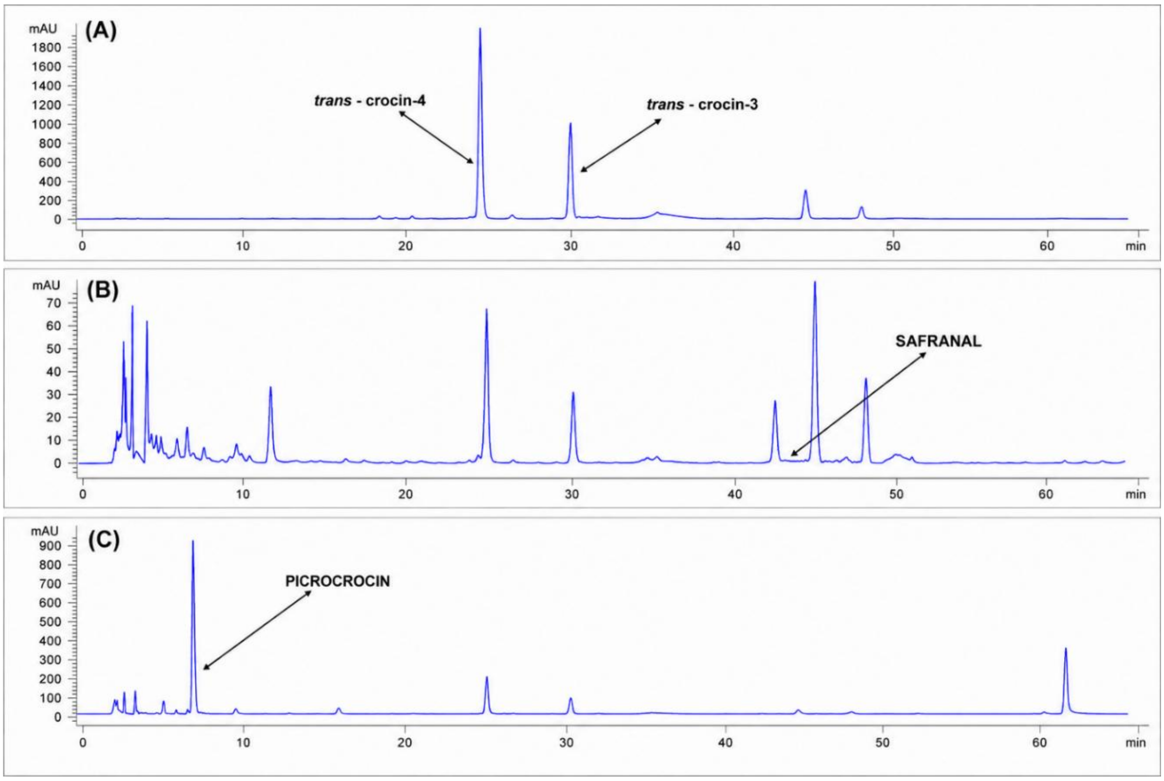

Figure. Certificate of Analysis

PHARMACTIVE
BIOTECH PRODUCTS
a natural difference

CERTIFICATE OF ANALYSIS

affron®

Scientific name:	*Crocus sativus*	Batch:	SAF-30695-PH
Country of origin:	Spain	Product code:	SAF0203107
Plant part used:	Stigmas	Manufacturing date:	December 2023
Extraction solvents:	Water	Expiry date:	December 2026
Composition:	Native extract and dextrin		
Appearance:	Dark orange powder with characteristic odor and taste		

ASSAY	METHOD	SPECIFICATION	RESULT
Identification (1)	HPLC-DAD/TLC	Positive	Conform
affron® profile (1)	HPLC-DAD	Positive	Conform
Lepticrosalides® (1)	HPLC-DAD	> 3.5%	4.19%
Bulk density (1)	EP (2.9.34)/USP <616>	> 0.3 g/mL	0.59 g/mL
Ash (1)	EP (2.4.16)/USP 37 <281>	< 10%	1.47%
Loss on drying (1)	EP (2.8.17)/USP 37 <731>	< 8%	3.26%
Particle size (1)	EP (2.9.12)/USP 34 <786>	250 µm/60 mesh	Conform
**MICROBIOLOGY**		EC 2073/2005 & EP 5.1.8*	
TAMC (1)	EP (2.6.12/2.6.13/2.6.31) USP 37 <61/62>	< 10 000 cfu/g	Conform
TYMC (1)		< 100 cfu/g	Conform
Enterobacteria (2)		< 100 cfu/g	Conform
L. monocytogenes (2)		Absent/25g	Conform
E. coli (2)		Absent/1g	Conform
S. aureus (2)		Absent/1g	Conform
Salmonella spp (2)		Absent/25g	Conform
**CONTAMINANTS** (2)		EC 1881/2006 & modifications	
Lead (Pb)	ICP-MS	< 3.0 ppm	Conform
Arsenic (As)		< 1.0 ppm	Conform
Cadmium (Cd)		< 1.0 ppm	Conform
Mercury (Hg)		< 0.1 ppm	Conform
PAH - BaP	GC-MS/MS	< 10 ppb	Conform
PAHs (Σ BaP, BaA, BbF, CHR)		< 50 ppb	Conform
Aflatoxin B1	HPLC-MS/MS	< 5 ppb	Conform
Aflatoxins (Σ B1, B2, G1, G2)		< 10 ppb	Conform
Melamine	HPLC-MS/MS	< 2.5 ppm	Conform
Pyrrolizidine alkaloids	HPLC-MS/MS	< 400 ppb	Conform
**PESTICIDES** (2)		EC 396/2005 & modifications	
Pesticides residues	HPLC & GC-MS/MS	According to regulation	Conform

- Packaging: food grade LDPE or PA/PE bags.
- Shelf life: 36 months if stored sealed in the original container at room temperature (<25ºC), sheltered from light and moisture (<60% RH).
- Control plan frequency: (1) analyzed on each production batch, (2) analyzed externally once a year, on the ingredient or the raw materials.
- *According to EP, products must meet the acceptance criteria or at least the maximum acceptable count tolerated described on this monograph.
- Natural variations in the raw material may lead to color variations from batch to batch but without affecting the quality and efficacy of the product.

PHARMACTIVE BIOTECH PRODUCTS
VAT #: ES B 85739860

HEADQUARTERS
Av. del Dr. Severo Ochoa, 37 Local 4J
Alcobendas 28108 Madrid (Spain)

R&D+QC LABORATORY
C/ Faraday, 7
28049 Madrid (Spain)

+34 911 123 848 - www.pharmactive.eu - info@pharmactive.eu

Pharmactive Biotech Products, SLU. - C/ Faraday, 7; 28049 Madrid, Spain. - Inscrita RM T27119 F 26, S 8, H M 488567, I/A 1 (30.10.09). CIF: B85739860.

References

Caballero-Ortega, H., Pereda-Miranda, R., & Abdullaev, F. I. (2007). HPLC quantification of major active components from 11 different saffron (Crocus sativus L.) sources. *Food Chemistry*, *100*(3), 1126–1131. https://doi.org/10.1016/j.foodchem.2005.11.020

Carmona, M., Zalacain, A., Sanchez, A. M., Novella, J. L., & Alonso, G. L. (2006). Crocetin esters, picrocrocin and its related compounds present in Crocus sativus stigmas and Gardenia jasminoides fruits. Tentative identification of seven new compounds by LC-ESI-MS. *J Agric Food Chem*, *54*(3), 973–979. https://doi.org/10.1021/jf052297w

Inarejos-Garcia, A. M., Prodanov, M., Rao, A., Garcés Rimón, M., & Raymond, J. (2017). *Empleo de un nuevo extracto de azafrán para la prevención de trastornos del estado de ánimo relacionados con la depresión* (Spain Patent No. https://patents.google.com/patent/ES2573542B1/en

Inarejos-Garcia, A. M., Prodanov, M., Rao, A., & Raymond, J. (2016). *Extracto de azafrán y su uso para la prevención de trastornos del estado de ánimo relacionados con la depresión* (Spain Patent No. https://patents.google.com/patent/ES2573542B1/es

Mena-Garcia, A., Herrero-Gutierrez, D., Sanz, M. L., Diez-Municio, M., & Ruiz-Matute, A. I. (2023). Fingerprint of Characteristic Saffron Compounds as Novel Standardization of Commercial Crocus sativus Extracts. *Foods*, *12*(8). https://doi.org/10.3390/foods12081634

Supplementary Table 1. Change in outcome measures from Week 0 to Week 12 (estimated marginal means) (PPS)

		Placebo (n = 38)						Saffron (n = 39)						p-value[b]	Cohen's D
		Week 0[a]	Week 4[a]	Week 8[a]	Week 12[a]	Change[b]	P-value[a]	Week 0[a]	Week 4[a]	Week 8[a]	Week 12[a]	Change[b]	P-value[a]		
DASS -D score	Mean	11.51	9.68	8.13	8.02	3.89	0.001	13.81	9.04	7.41	6.55	6.88	< .001	0.012	0.60
	SE	0.92	1.09	1.03	1.00	0.81		0.91	1.08	1.02	0.99	0.80			
RSE score	Mean	28.10	28.82	29.16	29.35	1.26	0.078	27.87	29.29	30.26	30.61	2.72	< .001	0.034	0.50
	SE	0.76	0.82	0.81	0.81	0.48		0.75	0.81	0.80	0.80	0.48			
SASA score	Mean	16.69	16.92	17.63	17.95	1.29	0.082	16.10	16.88	17.76	17.52	1.39	0.031	0.891	0.03
	SE	0.75	0.77	0.74	0.82	0.49		0.74	0.76	0.73	0.81	0.49			
PROMIS Sleep Disturbance (T-score)	Mean	61.03	56.14	55.81	54.67	6.08	< .001	59.98	53.81	52.98	53.46	6.80	< .001	0.632	0.11
	SE	0.89	1.05	1.15	1.13	1.06		0.88	1.04	1.14	1.11	1.05			
PROMIS Sleep-Related Impairment (T-score)	Mean	55.99	52.46	52.03	52.64	3.63	0.036	57.43	51.43	50.58	49.99	7.16	< .001	0.029	0.51
	SE	1.06	1.21	1.35	1.28	1.12		1.04	1.20	1.33	1.27	1.11			
Skin Age (yrs)	Mean	59.34	-	-	58.55	-0.60	0.526	58.72	-	-	57.85	-1.06	0.485	0.795	NA
	SE	0.92	-	-	1.16	1.12		0.91	-	-	1.15	1.11			

[a] P-values (within group) and estimated means are generated from repeated-measures ANOVAs adjusted for age and BMI.

[b] P-values and estimated means (change from W0 to W12) generated from univariate ANOVAs adjusted for age, BMI, and corresponding baseline value.

[c] P-value generated from an Independent-Samples Mann-Whitney U Test

PPS = Per-Protocol Set

Supplementary Table 2. Frequency of PGIC Responses at Week 12

	Placebo	Saffron	p-value*
Very Much Improved	0 (0.0%)	1 (2.5%)	0.630
Much improved	6 (15.0%)	7 (17.5%)	
Minimally improved	11 (27.5%)	11 (27.5%)	
No change	23 (57.5%)	19 (47.5%)	
Minimally worse	0 (0.0%)	1 (2.5%)	
Much worse	0 (0.0%)	1 (2.5%)	

* Chi-square test; PGIC = Patient Global Impression of Change

Note: Percentages calculated based on the number of participants who completed the study and provided PGIC ratings at week 12 (Placebo = 40; Saffron = 41)

Supplementary Table 3. Possibly or Probably Related AEs by Class and Term

**AE Class**	**Diagnosis or symptom**	**Placebo (n = 43)**	**Saffron (n = 43)**
Dermatological	**Number of participants**	**0 (0.0%)**	**1 (2.3%)**
	Itchy scalp	0 (0.0%)	1 (2.3%)
Gastrointestinal	**Number of participants**	**1 (2.3%)**	**1 (2.3%)**
	Constipation	0 (0.0%)	1 (2.3%)
	Abdominal distension	1 (2.3%)	1 (2.3%)
Neurological	**Number of participants**	**2 (4.6%)**	**1 (2.3%)**
	Worsened sleep	1 (2.3%)	0 (0.0%)
	Fatigue	1 (2.3%)	1 (2.3%)
Respiratory	**Number of participants**	**1 (2.3%)**	**0 (0.0%)**
	Shortness of breath	1 (2.3%)	0 (0.0%)
**Number of participants experiencing no treatment-related AEs**		**40 (93.0%)**	**41 (95.3%)**

*Some participants experienced more than one treatment-related AE

Supplementary Table 4. Frequency of PGATT Responses at Week 12

	**Placebo**	**Saffron**	**P-value***
EXCELLENT. I experienced no discomfort or adverse effects	34 (82.9%)	39 (97.5%)	0.086
GOOD. I experienced minimal discomfort/ side effects, but it did not interfere with my normal activities	6 (14.6%)	1 (2.5%)	
MODERATE. I experienced moderate discomfort/ side effects, and it had some effect on my normal activities	1 (2.4%)	0 (0.0%)	

* Chi-square test; PGATT = Patient Global Assessment of Tolerability to Therapy

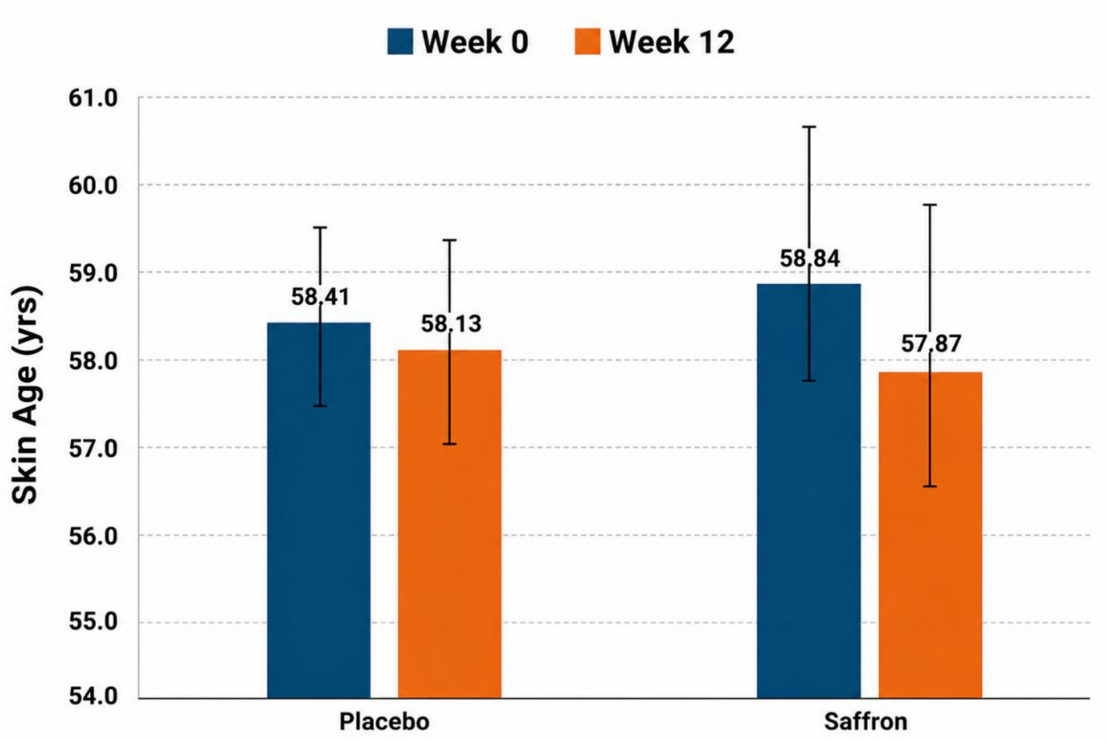

Supplementary Figure 1. Change in Skin Age from Week 0 to Week 12 (estimated marginal means) (FAS)

(Vertical bars represent +/- 1 standard error)
